# A mathematical model for the onset of avascular tumor growth in response to the loss of p53 function

**Published:** 2007-02-17

**Authors:** Howard A. Levine, Michael W. Smiley, Anna L. Tucker, Marit Nilsen-Hamilton

**Affiliations:** 1 Department of Mathematics; 2 Department of Biochemistry, Biophysics and Molecular Biology, Iowa State University, Ames, Iowa, 50011

## Abstract

We present a mathematical model for the formation of an avascular tumor based on the loss by gene mutation of the tumor suppressor function of p53. The wild type p53 protein regulates apoptosis, cell expression of growth factor and matrix metalloproteinase, which are regulatory functions that many mutant p53 proteins do not possess. The focus is on a description of cell movement as the transport of cell population density rather than as the movement of individual cells. In contrast to earlier works on solid tumor growth, a model is proposed for the initiation of tumor growth. The central idea, taken from the mathematical theory of dynamical systems, is to view the loss of p53 function in a few cells as a small instability in a rest state for an appropriate system of differential equations describing cell movement. This instability is shown (numerically) to lead to a second, spatially inhomogeneous, solution that can be thought of as a solid tumor whose growth is nutrient diffusion limited. In this formulation, one is led to a system of nine partial differential equations. We show computationally that there can be tumor states that coexist with benign states and that are highly unstable in the sense that a slight increase in tumor size results in the tumor occupying the sample region while a slight decrease in tumor size results in its ultimate disappearance.

## Introduction

There is solid evidence for the existence of a tumor suppressor gene known as p53. The *p*53 protein functions at several levels to control cell growth. It can inhibit the transcriptional activity of other proteins such as Sp1 and HIF-*α*, both of which activate growth factor (*GF*) gene expression [51, 50, 57, 60, 65]. It can also inhibit cell mitosis and regulate cell apoptosis [50]. In particular, one can view p53 as a tumor growth inhibitor and Sp1 as a tumor growth stimulator. (There are other cell-generated inhibitors and stimulaters but we shall confine our attention to these two.)

**Figure 1.**
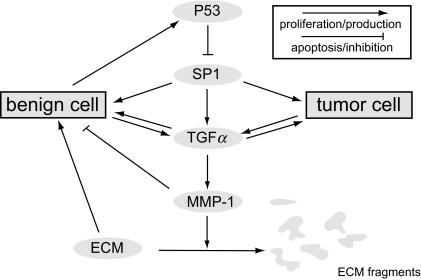
Biochemical schematics: This figure maybe regarded as the biochemical underpinning of our model. (Such figures are sometimes informally called “wiring diagrams” or “network diagrams”.) For example, benign cells express the wild type p53, which in its turn inhibits the transcription factor SP1 from initiating TGF *α* expression. Hence the blunt arrow leading from p53 to Sp1. No such inhibition is possible from the mutant p53 and hence is not shown in the figure or included in the model. Similarly, the double arrows express the fact that both cell types will secrete TGF*α* and in turn are influenced by this growth factor. The pathway from TGF*α* to the ECM proteins is really a schematic for the cell production of the matrix metallo-protein, which in its turn degrades the ECM via “standard” enzyme kinetics. Such diagrams, popular in the biochemical and molecular biology community, contain the seeds of systems of differential equations that begin with a consideration of the Law of Mass Action. In this sense, our model is in the spirit of [14] where the authors modeled the citric acid cycle in order to predict the onset of solid tumor growth in the micro environment.

The purpose of this paper is to present a biochemically based model for the onset of solid tumor formation. The model describes the onset of solid tumor formation with diffusion limited growth on a scale consistent with diffusion limited avascular tumor sizes. A schematic summary of the underlying biochemical pathway is given in Figure 1. This paper does not go beyond this point to treat the onset of vascularization due to the diffusion of the carcinogenic waste products of tumor necrosis (tumor angiogenesis). This idea is not new in the sense that in [14] the authors used the kinetics of cell metabolism, to model the growth of tumor cells in the micro environment.

It is not our intention to assert that this is the *only* way avascular tumor growth can arise from gene mutation.

Among many examples are cancers such as lymphoma that are driven by an overexpressed and mutated MYC gene [7, 32]. The wild type MYC protein both inactivates the cell cycle by suppressing p21 and suppresses apoptosis by inducing BIM. The mutant forms of the MYC protein found in these cancer fail to induce BIM [see ref below]. This pathway runs in parallel with the p53 pathway, which is also activated by MYC ([32]). The mathematical modeling of this biochemical pathway is very similar to the the pathway we consider here in many respects.

*The goal here is to combine the loss of p53 regulatory function in benign cells with the consequent increase in the rate of benign cell loss (via mutation into tumor cells) and the effect this mutation has on the rate of formation tumor cells in order to model solid tumor growth in the micro environment*. The model attempts to duplicate nature in the sense that, in the model, it is assumed that a few benign cells undergo a mutation by which they lose the ability to express the wild type p53 gene. More precisely, it is assumed that a few benign cells undergo a mutation by which there is a loss of wild type p53 expression over a small spatial region for a fixed time period. (Following the usual biochemical convention, when we refer to p53, we will only mean the wild type because the mutant type does not regulate Sp1 (and hence growth factor) for one of several reasons including (a) an incorrect protein transcribed from the mutant RNA, (b) no mutant p53 at all or (c) a mutant p53 with no suppresser capability.) This loss of tumor suppressor function will, in the model, result in excess Sp1 production. Sp1 functions as a transcription factor for, among other genes, the gene for MMP-1, a protease that is known to degrade tissue structural proteins such as collagen. This idea of genetic instability is not new (see [69]) for example. However, we think the modeling approach, as it combines fundamental biochemistry with chemotaxis and modification of classical competition dynamics, is novel.

We begin with an initial state in which we have a region that consists only of benign cells and matrix. This state constitutes a stationary solution for a dynamical system based upon the biochemistry described above in the absence of loss of function. Our results show that the length of the period of mutation (the time interval over which the loss of function occurs), the spatial size (the volume over which loss of function occurs) and the intensity of the mutation (percentage of cells in which loss of function occurs) determine whether the tumor cells take over the region of interest. In biological terms, the period of the mutation refers to the period during which the tissue is exposed to a mutagen, the spatial size refers to the volume of tissue that is exposed to the mutagen, and the intensity of the mutation refers to the concentration and potency of the mutagen. For example, the frequency of p53 loss of function in the skin is influenced by the period of exposure to uv light, the area of skin exposed to the light and the intensity and spectrum of the light.

Mathematically, the loss of function is modeled by the simple device of lowering the effective p53 rate constant for a specified portion of time and over a specified region in the benign cell region for a specified percentage (intensity). (See equation (2.1) and notice the modification in the coefficient *kp* in equation (2.2).) The mutation in cell type is then modeled by a corresponding loss of benign cell growth rate coupled with an increase in tumor cell cell growth rate whenever the concentration of p53 falls below a threshold level. (See equation (3.5) and notice the last term on the right hand side of each rate law.)

In particular, the model shows that:

1. When the mutation is sufficiently intense, widespread or of sufficiently long duration, the malignant cell mass that develops during the loss of function period contains sufficient malignant cell mass to take over the region under consideration.
2. On the other hand, if the mutation is insufficiently intense, very local or of insufficient duration, the initial transfer of some cells from the benign state into the tumor state induced by the mutation is not sustainable and the small tumor perturbation dies out, with the system returning to its initial state.
3. The model postulates that if the intensity is small enough, and the region over which the mutation occurs is fixed, the duration time can never be long enough to initiate a growing tumor.
4. The model postulates the existence of tumor states that can coexist with benign states for very long times (i.e. form coexisting quasi-steady states.)

Some comments on the mathematical literature: There is a large literature on the mathematical modeling of various aspects of solid tumor growth. See [1, 2, 3, 4, 13, 12, 15, 35, 37, 62] for some representative examples. Some of this literature reflects the controversial nature of modeling solid tumor growth. See for example [30] and the rebuttal [58]. There is also an extensive literature concerned with the spontaneous remission of solid tumor growth, i.e. tumors that disappeared without treatment of any kind. A key word search on the phrase “spontaneous remission or regression of cancer” in PubMed led to over 2200 screens of articles. Many of the articles listed were case studies of various identified cancers. A few dealt with various mathematical aspects of the problem [36, 70]. We suggest that our results demonstrate one possible mechanism for this remission.

There are a number of papers dealing with competition models and Lotka-Volterra systems, as systems of ordinary differential equations and as systems of reaction diffusion equations, see for example [11, 18, 21, 22, 19, 20, 34, 38]. However we could find no papers in which these systems were combined with Keller-Segal type equations of chemotaxis. (We did a keyword search on MathSci-Net and found no matches for “competition species” AND “chemotaxis” or “competition species” AND “Keller-Segal”.) Our model does combine chemotaxis for cell movement together with the “competition species” notion for competing species. Given the dearth of literature on these combined notions, perhaps we can claim some novelty for our model. *It is not only mathematically naive but also false, to draw the inference that conclusions about the dynamical behavior of solutions of a system ordinary differential equations such as the Lotka-Volterra system for competition systems, will lead to valid conclusions about analogous systems of partial differential equations when these equations are combined with diffusion terms or diffusion with chemotactic terms. Turing instability is probably the classical example of the failure of the stability properties of nonlinear ordinary differential equations to be inherited by systems of partial differential equations with the nonlinearities arising from the ode system. See also the two very nice papers* [45, 63] *for alternate approaches to this issue.*

Here is an outline of the remainder of the paper. Sections 2–8 constitute the main body of the paper.

- Section 2: We present the biochemical and cell biological underpinnings of the model. The details of the biochemistry are set forth in Appendix A.
- Section 3: We discuss the cell movement equations. Here we use the notion that cells move up or down chemical gradients (chemotaxis) together with the idea that mitosis of each cell type interferes with the mitosis of the other.
- Section 4: We set forth the mechanistic equations that define the notion of diffusion limited tumor growth. That is, the entire system is governed by the consumption of nutrients delivered to the tumor region via diffusion. Waste products are generated as a consequence of protein degradation and cell apotosis. Because we do not use the waste products further as sources for tumor growth, we ignore their evolution here.
- Section 5: We give the appropriate boundary conditions and initial conditions for the problem dynamics.
- Section 6: We discuss the dynamics predicted by a subsystem of ordinary differential equations.
- Section 7: We describe how the normalizing constants are selected in order to nondimensionalize the system and how the initial steady state is found. First the system (without mutational considerations) is modified in such a way that constant solutions of the modified system can be found explicitly. These constant values are used to non-dimensionalize the full problem. By using the constant values as initial conditions for the system (without mutational considerations) the putative non-constant, benign solution is obtained (numerically) as the time independent limit of the system.
- Section 8: We present the results of our simulations and our conclusions. We consider our problem with spherical symmetry to keep the computational complexity to a minimum.
- Appendix A: We give a brief overview of the molecular biology and the resultant chemical kinetics upon which we base our model.
- Appendix B: We record the numerical values of the constants we used in the simulations.

The reader is warned that quite a bit of mathematics lies ahead. We do not apologize for this, since in order to use mathematics to describe a complex biochemical process, one might expect that the mathematical description itself might likewise be involved. However, to make the reader’s task somewhat more palatable, we offer the following observations. Nothing more complex than elementary enzyme kinetics, mass action and Fick’s law is involved in the description of the dynamics of the time evolution of the protein and nutrient concentrations as well as the local cell population densities. The nutrient equation controls the flow of nutrients reaching every point of the extracellular matrix.

## Notation and Kinetics

We consider the interplay among the proteins p53, GF the transcription factor, Sp1, the extra-cellular structural proteins *F*, and the matrix metalloproteinase, MMP-1 as described in Figure 1. In this model, it is assumed that transport of subcellular species such as growth factor, p53 etc. is passive, i.e. is controlled by cell movement. We imagine a region *D* in two or three dimensional space. Throughout, **X** = (*x*, *y*) or **X** = (*x*, *y*, *z*) according to the dimension in which *D* lies. We use the notation in Table 1. We have eleven species, nine of which are chemical, two of which are cellular. (In the sequel the constants *N_B_*, *N_T_* denote the carrying capacity densities for benign and tumor cells respectively.) Throughout this paper we adopt the convention that the Greek *μ* is reserved for decay (turnover) constants while *ν* is reserved for equilibrium constants.

We begin with p53. A relationship for the rate of formation of this transcription factor based on the number of benign cells present and level of resources is given by

(2.1) $$\frac{\partial p}{\partial t}=\frac{k_{p}Y\;(\text{x},t)}{K_{p}+Y\;(\text{x},t)}\frac{\eta_{B}\;(\text{x},t)}{N_{B}}-\mu_{p}p\;(\text{x},t).$$

The assumption here is that only benign cells express the wild type gene, wherever they appear in the tissue. The cell density has been re-scaled so that *k_p_* now includes the maximum possible benign cell density as a factor. (In Appendix A, we give a rationale for the term $\frac{k_{p}Y\;(\text{x},t)}{k_{p}+Y\;(\text{x},t)}\frac{\eta_{B}\;(\text{x},t)}{N_{B}}$ based on chemical kinetics.) We refer to (2.1) as the equation of p53 evolution **without loss of function.**

In order to express the idea of evolution of p53 **with loss of function**, consider a subregion *D'* ⊂ *D*, a time interval (more generally, several time intervals) *I* = (*T*_1_, *T*_2_) and a function Ψ(**x**, *t*) with support in *D'* × *I* and having values in (0,1]. A mutation is said to occur in the region *D'* ⊂ *D* over a time interval *I* if there has been a loss of p53 expression in the set *D'* × *I*. That is, we replace (2.1) by

(2.2) $$\begin{array}{l} \frac{\partial p}{\partial t}=\frac{k_{p}\;[1-\Psi \;(\text{x},t)\;]Y\;(\text{x},t)}{K_{p}+Y(\text{x},t)}\frac{\eta_{B}(\text{x},t)}{N_{B}}\\ \;\;\;-\mu_{p}p(\text{x},t). \end{array}$$

We refer to this equation as **the evolution equation for p**53 **with loss of function** or as **the mutated p**53 **equation**. The function Ψ is called the loss of function coefficient. The intensity of the loss of function is defined as

(2.3) $$I=\frac{1}{V_{D^{\prime }}L(I)}\int_{D^{\prime }}\int_{I}\Psi (\text{x},t)\text{dtd}\text{x}$$

where *V_D_*_′_ *L*(*I*) is the product of the volume of *D*′ and the length of the time interval over which the loss of function occurs. Notice that 0<I≤1. The strength of the mutation is defined as

(2.4) $$S=V_{D^{\prime }}L(I)I.$$

Corresponding to such a loss of function coefficient will be a drop (pointwise) in p53 concentration. When this concentration falls below a certain level, *p_c_* say, some benign cells will become tumor cells. The model is not reversible. That is, tumor cells can only disappear via apoptosis. For the time being, it is enough to remark that each equation (2.1), (2.2) will play a role as part of a larger system. The system consisting of (2.1) and the remaining dynamics (discussed below) will be used to compute a non constant steady state in which there are no tumor cells present. This steady state will then form the initial condition for the system consisting of (2.2) and the dynamics discussed below.

**Remark 1.** There is experimental evidence that *p*53 in normal tissue is much more stable than in malignant tissue. For example, in [6] it was reported that although this protein has a half life of about three hours in normal mammary epithelial cells, it was approximately 15 minutes in E5 immortalized cells, i e. cells that were immortalized by the E6 gene of HPV-16, the human papilloma virus commonly associated with cervical cancers. We have not included this observation as it is clear intuitively that its effect should drive up the expression of active Sp1 and hence the level of growth factor over what we already have computed in the regions where tumor cells are present.

From the literature [51] we know that p53 inhibits the transcription factor Sp1. We assume that this occurs via the equilibrium:

(2.5) $$T_{r}^{a}+P\overset{V_{e}^{s}}{⇄}T_{r}^{i}$$

where $T_{r}^{a}$, $T_{r}^{i}$ represent the active and p53 inhibited form of Sp1. We write:

(2.6) $$\begin{array}{l} \frac{\partial_{S_{B}}}{\partial t}=\frac{k_{s}Y\;(\text{x},t)}{K_{s}+Y\;(\text{x},t)}\left(\frac{\eta_{B}(\text{x},t)}{N_{B}}\right)\\ \;\;\;-\mu_{s_{B}}S_{B}\;(\text{x},t) \end{array}$$

for Sp1 growth and decay in benign cells. In tumor cells we write

(2.7) $$\begin{array}{l} \frac{\partial_{S_{T}}}{\partial t}=\frac{k_{s}Y(\text{x},t)}{K_{s}+Y(\text{x},t)}\left(\frac{\eta_{T}(\text{x},t)}{N_{T}}\right)\\ \;\;\;-\mu_{s_{T}}S_{T}(\text{x},t) \end{array}$$

where *μ*_*s*_*T*__ ≤ *μ*_*s*_*B*__ since the decay rate (the turnover rate) for Sp1 in benign cells is larger than that for tumor cells in general. We have, in terms of the notation in Table 1, *for benign cells only*

(2.8) $$s_{B}^{a}+s_{B}^{i}=s_{B},s_{B}^{i}=v_{e}^{s}s_{B}^{a}p,$$

and hence

$$s_{B}^{a}\;=\;\frac{s_{B}}{1+v_{e}^{s}p}.$$

Before proceeding further, we need some terminology. By a “switch” we mean a Heaviside function, i.e. a function *H*(*x*) which is zero if *x* < 0 and one if *x* > 0.^1^ In the single cell, a switch is either on or off. In a population model such as this one, the transition from “off” to “on” is actually smooth and “smooth” versions of the Heaviside function were employed in the simulations. For example, one could use *H*(*x, K*) = max{*x^m^*/(*K* + |*x*|*^m^*),0)} where *K* > 0 and *m* is an odd positive integer so that *H*(*K*^1/*m*^*, K*) = 1/2. Alternatively *H*(*x,m*) = max{0, 1 − exp(−*mx*)} and *H*(ln 2/*m*,*m*) = 1/2.

We turn to the action of the growth factor. We consider not only the action of the transcription factor Sp1 on GF synthesis but also the degradation of growth factor during the course of the cell synthesis of MMP-1. The synthesis and degradation of growth factor is assumed to be regulated by Sp1 according to the discussion in the appendix. Thus we write:

(2.9) $$\begin{array}{l} \frac{\partial v}{\partial t}=\frac{k_{v}Y(\text{x},t)}{K_{v}+Y(\text{x},t)}(H(s_{B}^{a})\frac{\eta_{B}(\text{x},t)}{N_{B}}\\ \;\;+H(s_{T})\frac{\eta_{T}(\text{x},t)}{N_{T}})-\mu_{v}v(\text{x},t). \end{array}$$

Clearly, as the concentration of p53 falls, more inhibited Sp1 in benign cells is converted to the active form. This causes an increase in growth factor production.

A simplified scenario for the GF role in the production of MMP-1 might be the following: Once the cell has expressed a molecule of GF and released it to the ECM, the molecule binds to a GF receptor (R) and initiates signaling via a MAP-kinase pathway to induce transcription of the MMP-1 gene with the eventual translation to create the protease. A simplified mechanism for this is given in [44]. That mechanism, in its turn, is a bit more complicated than we need for our purposes here. Instead we use:

(2.10) $$\begin{array}{c} V+R\overset{k_{on}^{r}}{\underset{k_{off}^{r}}{⇄}}\{VR\}\\ Y+\{VR\}\overset{k_{m}^{r}}{\to }nM+V^{\prime }+R \end{array}$$

where *V*′ denotes the products of GF degradation. Michealis-Menten kinetics for this system yields, assuming *V* is in excess so that the concentration of the intermediate {*VR*} is constant:

$$\frac{d[M]}{dt}\;=\;\frac{nk_{on}^{r}k_{off}^{r}[V][Y][R]}{k_{off}^{r}\;+\;k_{m}^{r}[Y]}$$

The number *n* is the cell amplification factor for MMP-1. It is thought to be fairly large and is a consequence of the amplification properties of the MAP-kinase signaling pathway and transcriptional response to growth factor. It is not constant, but depends upon the local concentration of growth factor, first increasing and then decreasing with growth factor concentration [61].

Likewise, there is a corresponding loss of growth factor that follows from the kinetics (2.10), namely

$$\frac{d[V]}{dt}\;=\;\frac{k_{on}^{r}k_{off}^{r}[V][Y][R]}{k_{off}^{r}\;+\;k_{m}^{r}[Y]}$$

In the mechanism (2.10), we need to distinguish carefully between the receptors on the tumor cells and the receptors on the benign cells. Relating receptor density to cell density, and taking into account the transcription of GF as well as its decay and conversion to MMP-1, we may write:

(2.11) $$\begin{array}{l} \frac{\partial \nu }{\partial t}=-\frac{k_{v}Y\;(\text{x},t)}{K_{v}+Y(\text{x},t)}(H\left(s_{B}^{a}(\text{x},t)\right)\frac{\eta_{B}(\text{x},t)}{N_{B}}\\ \;\;\;+H(s_{T}(\text{x},t))\frac{\eta_{T}(\text{x},t)}{N_{T}})-\mu_{v}v(\text{x},t)\\ \;\;\;-\frac{K_{\text{cat}}^{r}v(\text{x},t)Y(\text{x},t)}{K_{m}^{r}+Y(\text{x},t)}\left(\frac{\eta_{B}(\text{x},t)}{N_{B}}+\frac{\eta_{T}(\text{x},t)}{N_{T}}\right). \end{array}$$

In turn, growth factor induces the cellular expression of MMP-1:

(2.12) $$\begin{array}{l} \frac{\partial m}{\partial t}=Y(\text{x},t)\frac{nK_{\text{cat}}^{r}v(\text{x},t)}{K_{m}^{r}+Y(\text{x},t)}(\frac{\eta_{B}(\text{x},t)}{N_{B}}\\ \;\;+\frac{\eta_{T}(\text{x},t)}{N_{T}})-\mu_{m}m(\text{x},t). \end{array}$$

We have neglected the diffusion of growth factor in (2.11) because its half life is short in tissues.

Next we consider the rate equation for collagen/fibronectin degradation. It has been established experimentally that some tumor cell types have a limited ability to express fibronectin relative to benign cells [16, 54, 24, 64]. We assume that this rate is a small percent, *ɛ_f_*, of the rate of production of benign cells. Moreover, the molar rate of production of collagen/fibronectin must not only depend upon the local cell density but also upon the local concentration of resources, up to some saturable limit. To reflect these ideas, we write^2^:

(2.13) $$\begin{array}{l} \frac{\partial f}{\partial t}=\frac{\left(4/T_{f})Y(\text{x},t\right)}{K_{f}+Y(\text{x},t)}f(\text{x},t)(1-\frac{f(\text{x},t)}{f_{M}}\\ \;\;\;\left(\frac{\eta_{B}(\text{x},t)}{N_{B}}+\varepsilon f\frac{\eta_{T}(\text{x},t)}{N_{T}}\right)\\ \;\;\;-\frac{K_{cat}^{f}m(\text{x},t)f(\text{x},t)}{K_{m}^{f}+f(\text{x},t)}. \end{array}$$

We have written 4/*T_f_* as the time constant at saturation for convenience. In this form, at saturation and low concentrations of *f*, the doubling time for *f* is *T_f_* ln 2/4. The factor of four is included since then 4*x*(1 − *x*) has a maximum value of unity.

## Cell Movement

In order to track the movement of cells consider again the kinetics that follow from (2.10). The law of mass action when applied to the rate equation for the receptor density would yield ∂[*R*]/∂*t* = 0 in consequence of the Michealis-Menten hypothesis that [*RV*] is nearly constant. However, the issue is somewhat more complicated in this case because: (1) the receptor distribution is tied to the cell membrane movement; (2) the cell movement is dependent on the local concentrations of growth factor, enzyme and ECM protein in a chemotactic (and chemokinetic) as well as haptotactic manner rather than being completely random; and (3), the cells themselves undergo mitosis and apoptosis (programmed cell death). However, we can relate cell density to receptor concentration via the relationship [R](**x**, *t*) = *cN*(**x**, *t*) where the left hand side has the units of micro molarity (micro moles per liter) say while the cell density *N* is expressed in cells per liter so that the constant has units of micro moles per cell. Then a rate for [*R*] is proportional to a rate for *N* and we can consider cell movement rate laws in their own right coupled to the protein movement laws.

Suppose, for the moment, that *N* is the density of one of the two cell types under consideration here. From the continuity equation in the absence of sources or sinks (mitosis and cell death)

(3.1) $$\partial_{t}N=-\vec{\nabla }.\;\vec{J}$$

where $\vec{J}$ is the local flux of cell density at (**x**, *t*).

Fick’s law is then modified so that the flux of cell density will be influenced by the gradient in protease density *m*(**x**, *t*) (chemotaxis), and gradients in collagen density *f* (**x**, *t*) (haptotaxis). These notions mean that each biochemical species influences the flux of the cells through a term that is proportional to the cell density and that depends on the gradient of the species, vis:

(3.2) $$\begin{array}{l} \vec{J}=D(N)\{-\vec{\nabla }N+[M(m,f)\vec{\nabla }m\\ \;\;+F(m,f)\vec{\nabla }f]N\} \end{array}$$

where *M*, *F* are some phenomenological functions of (*m*, *f*). These functions, sometimes called the chemotactic sensitivity functions, determine the influence of the specific species on the flux of cell densities. For example, where *M* > 0 the gradient of protease opposes the cell density gradient while where *M* < 0, it assists that gradient. If one makes the assumption that the vector (*M*, *F*) = ∇[*T*(*m*, *f*)] where ∇ is now the gradient in the variables (*m*, *f*), for some potential function *T*^3^, writes T(*m*, *f*) ≡ exp(*T*(*m*, *f*)) and uses this in the resulting flux vector, then the cell movement equation (without sources and sinks) takes the form

(3.3) $$\frac{\partial N}{\partial t}=\nabla \cdot \left\{D_{N}\left[N\nabla 1\text{n}\left(\frac{\text{N}}{𝔗\;(m,f)}\right)\right]\right\}.$$

This is perhaps a strange way to write the equation for chemotactic cell movement. However, the meaning is clear. It says that near steady state, “*N* should follow 𝔗(*m*, *f*)”, an observation of Hans Weinberger (private communication).

The function 𝔗 (*m*, *f*) is called the probability transition function (PTF). We can understand its meaning at the intuitive level if we consider the stationary (time independent) version of the equation. Suppose *N*(*x*), *m*(*x*), *f* (*x*) are smooth functions connected by the relationship *N(x)* = λ 𝔗 (*m*(*x*), *f* (*x*)) for some constant λ. Then it is clear that *N*(·) must be a stationary solution of (3.3) and we say that “the solution *N* follows𝔗”.

Another way of looking at (3.3) is from the point of view of “cellular free energy”. That is, one views 𝔗 as a correction factor for chemotaxis/haptotaxis in the associated “cellular potential” here corresponding to the cell density. The “cellular potential” is defined as *ρ*(*N*) = −*D* ln (*N*/*N*_0_) where *N*_0_ is the “concentration” in some reference state. Without the correction factor, using Fick’s first law, *N_t_* = −∇ *J* where *J* = −*DN* ∇ *ρ* (*N*) we obtain the ordinary diffusion equation for cell density. We can think of the cellular potential in much the same way as the chemist thinks of chemical potential for the isothermal change in free energy. Then we view *N*/𝔗(*m*, *f*) in much the same way as a chemist thinks of fugacity (mole fraction times pressure) at zero pressure in non-ideal gas dynamics or activity (which replaces ion concentration) in ionic solutions at finite dilution. See books on chemical thermodynamics such as [68] for more information. (The authors thank James Keener for bringing to our attention this interpretation of the appearance of the logarithm in (3.3).)

The choice of 𝔗(*m*, *f*) is phenomenological. Other possible dependencies for the probability transition rate function 𝔗 are certainly possible. For example, 𝔗(*m*, *v*, *f*) is a possibility. Any other external biochemical variable for which it is known that cells respond in a chemotactic/haptotactic manner may also be added to the argument list. For example it has been observed that endothelial cells will move up a protease gradient, but if the concentration of protease is too high, the enzyme will kill the cell, causing the cessation of movement. Likewise, EC cannot move through a matrigel bed if the density of matrigel is too high nor can they move along a surface unless there is some ECM protein such as collagen or fibronectin onto which it may attach its pseudopodia. Experimental evidence is given in [10]. These two qualitative observations suggest that this function should be biphasic in each variable (i. e. first increasing and then decreasing in each variable). The precise form we use in our simulations is described in Appendix B.

When we have two cell types present whose cell densities are denoted by *η_B_*, *η_T_*, say, (3.3) must be replaced by a system. One question that arises naturally is how to model the preference for cells of different types to fill regions vacated by dead cells. Generally speaking, as cells move, they consume the resources that are responsible for supplying the energy for cell movement. As this energy is exhausted, cell movement slows.

Additionally, the cells will behave differently under “crowding” conditions. That is, we expect the clumping of benign cells to slow their movement and push them into *G*_0_, whereas tumor cells, which do not enter *G*_0_, are more aggressive and not as influenced by the effects of crowding. In both cases, we expect movement to decrease as cell concentration rises, but we expect the rate of decrease of movement to be more rapid for benign cells than for tumor cells. Although we did not use this in our simulations, the model allows for this possibility. These ideas are much broader than we employ here [52]. Here we simply include the cell densities themselves as a part of the “chemotactic” sensitivity function and write

(3.4) $$\begin{array}{l} \frac{\partial \eta_{B}}{\partial t}=\nabla \cdot [D_{B}(\eta_{B},\eta_{T})\eta_{B}\nabla \\ \;\;\;\text{ln}\;\left(\frac{\eta_{B}}{{𝔗}_{B}(\eta_{B},\eta_{T},m,f)}\right)],\\ \frac{\partial \eta_{T}}{\partial t}=\nabla \cdot [D_{T}(\eta_{B},\eta_{T})\eta_{T}\nabla \\ \;\;\;\text{ln}\left(\frac{\eta_{T}}{{𝔗}_{T}(\eta_{B},\eta_{T},m,f)}\right)]. \end{array}$$

where we have omitted mitotic and apoptotic effects for the moment.

We next consider the issues of mitosis and apoptosis (sources and sinks) that must be included in both cell movement equations. These are viewed as forcing terms and are somewhat easier to explain. If we assume that the quantities of growth factor produced are close to the background rates, then cell proliferation rates may be taken to be nearly independent of growth factor. However this is not always true, especially in the case of angiogenesis. See [61] and [43] for an application of this fact.

It is known that p53 is a regulator of cell mitosis and proliferation. In particular, it enhances apoptosis and inhibits mitosis [50]. We make the assumption here that the distinguishing features between tumor cells and benign cells are the following:

1. A drop in *p*53 concentration below a critical value in benign cells results in the change of type (a mutation) of such cells to the malignant state. It is assumed that this occurs at a rate proportional to the benign cell density and is controlled by a switching mechanism. That is, the rate is proportional to *H*(*p_c_* −*p*)*η_B_*. The proportionality constant is denoted by λ*_tr_*. Here *p_c_* is a background critical value required to maintain a steady state population consisting only of benign cells in the region in question. *The transfer is one way only; it allows for the growth of tumor cells at the expense of benign cells by permitting the conversion of the latter to the former through a modification in their respective growth rates.*
2. The apoptosis to mitosis ratio of benign cells is higher than the corresponding ratio for tumor cells since the latter do not express p53 and the former do. (See [59] for some simple mathematical models of tumor growth based upon differing mitotic and apoptotic rates for tumor cells versus benign cells. See also the discussion in [7, 32].)
3. Both *D_B_*, $D_{T}$ are constant.
4. For * = *B*, *T*, the sensitivity functions 𝔗_*_(*η_B_*, *η_T_*, *m*, *f*) can be written as products 𝔗_*_(*m*, *f*) ψ_*_(Z) where *Z* = *η_B_*/*N_B_* + *η_T_*/*N_T_* is the volume fraction of a given region occupied by cells. From physical considerations, we have 0 ≤ *Z* ≤ 1. (A simple formal argument using the logistic equation for total cell growth, namely *dZ/dt* = *cZ*(1 −*Z*) convinces us that if 0 < *Z*(0) < 1, then *Z*(*t*) remains in this interval. In the general case one can argue from the maximum principle.) In reality, *Z* ≤ *Z_M_* < 1 where 1 − *Z_M_* is the volume fraction of the extracellular region between the cells. However, we shall ignore this volume fraction in the model.

Based on the above considerations, the cell movement equations take the form:

(3.5) $$\begin{array}{l} \frac{\partial \eta_{B}}{\partial t}=D_{B}\nabla \cdot \left[\eta_{B}\nabla \text{ln}\left(\frac{\eta_{B}}{{𝔗}_{B}(m,f)\Psi_{B}(Z)}\right)\right]\\ \;\;\;+[H(v-v_{B})\frac{\lambda_{B}Y}{\kappa_{B}+Y}\\ \;\;\;\left(1-\frac{\eta_{B}}{N_{B}}-L_{T}\frac{\eta_{T}}{N_{T}}\right)-\mu_{B}]\eta_{B}\\ \;\;\;-\lambda_{tr}H(p_{c}-p)\eta_{B},\\ \frac{\partial \eta_{T}}{\partial t}=D\nabla \cdot \left[\eta_{T}\nabla \text{In}\left(\frac{\eta_{T}}{{𝔗}_{T}(m,f)\Phi_{T}(Z)}\right)\right]\\ \;\;\;+[H(v)\frac{\lambda_{T}Y}{\kappa_{T}+Y}\\ \;\;\;\left(1-\frac{\eta_{T}}{N_{T}}-L_{B}\frac{\eta_{B}}{N_{B}}\right)-\mu_{T}]\eta_{T}\\ \;\;\;+\lambda_{tr}H(p_{c}-p)\eta_{B.} \end{array}$$

The factor *H*(*v* – *v_B_*) in the first of (3.5) says that when *v* > *v_B_* benign cells proliferate, while when the growth factor is below this level, they cannot. The factor *H*(*v*) in the second of (3.5) has a similar meaning for tumor cells (with *v_T_* = 0). These two factors reflect the fact benign cells are generally quiescent (in *G*_0_) whereas tumor cells are usually constantly passing through the cell cycle. The threshold *v_B_* is intended to model the lower proliferative response of benign cells to growth factor than that of tumor cells. Unfortunately, the number *v_B_* is unknown. Therefore, by taking *v_B_* = 0, in the model, we are giving the benign cells a greater survival advantage than they would ordinarily be expected to possess. The qualitative form of the results we give below will be unchanged by taking *v_B_* > 0. The factors *λ_B_Y*/(κ*_B_* + Y) and *λ_T_* Y/(κ*_T_* + Y) are included to reflect the idea that when nutrient levels are low, cell population production is low, but, as the nutrient levels rise, the population growth rates approach a saturation level given by the constants *λ_B_*, *λ_T_*.

The factors *L_T_*, *L_B_* are included as in [27, 28]. We refer to them as mitosis inhibition or apoptosis enhancement factors as they can be viewed as either inhibiting the former or enhancing the latter. (We have written the equations as though they inhibit the mitosis.) In general, we expect that *L_T_* ≥ *L_B_*, that is tumor cells influence the benign cell net proliferation rate much more than vice-versa. This clearly gives the proliferation advantage to tumor cells. However, it does not imply that under all circumstances, the tumor cells will take over the region of interest from the benign cells.

## Mass balance equations

In this model, nutrients function directly to support cell production of p53, Sp1, GF and MMP-1. These proteins also decay and are transported out of the region *D* by molecular diffusion (in the absence of a vasculature). Additionally, tumor and benign cells are undergoing mitosis and apoptosis in this region while collagen/fibronectin is being generated by the cells and degraded by MMP-1.

For example, in the rate equation for p53, resources are being consumed at a rate dictated by the first term on the right of (2.1) while the products of protein decay are being generated by the second term on the right of that equation. At steady state, as for example, in the healthy tissue, these two terms agree, otherwise they do not.

We have the mass rate loss for the resources:

(4.1) $$\begin{array}{ll} \frac{\partial Y}{\partial t}= & \nabla \cdot \;[D_{Y}\nabla Y]\\ & -Y\;(\text{x},t)\;[\frac{k_{p}}{K_{p}+Y\;(\text{x},t)}+\frac{k_{s}}{K_{s}+Y\;(\text{x},t)}\\ & \;\;\;+\frac{k_{v}H\;\left(S_{B}^{a}\;(\text{x},t)\right)}{K_{v}+Y\;(\text{x},t)}]\frac{\eta_{B}\;(\text{x},t)}{N_{B}}\\ & -Y(\text{x},t)\;[\frac{k_{s}}{K_{s}+Y\;(\text{x},t)}\\ & \;\;\;+\frac{k_{v}H\left(s_{T}\;(\text{x},t)\right)}{K_{v}+Y\;(\text{x},t)}]\frac{\eta_{T}\;(\text{x},t)}{N_{T}}\\ & -Y\;(\text{x},t)\;\{\frac{nK_{cat}^{r}v\;(\text{x},t)}{K_{m}^{r}+Y\;(\text{x},t)}[\frac{\eta_{B}\;(\text{x},t)}{N_{B}}\\ & \;\;\;+\frac{\eta_{T}\;(\text{x},t)}{N_{T}}]+\frac{\left(4\;/\;T_{f}\right)}{K_{f}\;+\;Y\;(\text{x},t)}f\;(\text{x},t)\\ & \;\;\;\left(1-\frac{f\;(\text{x},\;t)}{f_{M}}\right)\left[\frac{\eta_{B}\;(\text{x},t)}{N_{B}}+\varepsilon_{f}\frac{\eta_{T}\;(\text{x},\;t)}{N_{T}}\right]\\ & \;\;\;+[\ell_{B}\frac{\lambda_{B}H\;(v)}{\kappa_{B}+Y\;(\text{x},t)}\left(1-\frac{\eta_{B}}{N_{B}}-L_{T}\frac{\eta_{T}}{N_{T}}\right)\eta_{B}\\ & \;\;\;+\ell_{T}\frac{\lambda_{T}H\;(v)}{\kappa_{T}+Y\;(\text{x},t)}\left(1-\frac{\eta_{T}}{N_{T}}-L_{B}\frac{\eta_{B}}{N_{B}}\right)\eta_{T}]\}\\ & \equiv \nabla \cdot [D_{Y}\cdot \nabla \;Y]\\ & -Y\;\left(\text{x},t\right)\;𝔖\;(Y,f,s_{B}^{a},s_{T},v,\eta_{B},\eta_{T}) \end{array}$$

where 𝔖 is the (positive) coefficient of *Y* in the preceding line. The constants *ℓ_B_*, *ℓ_T_* are conversion factors that permit us to convert the units of cells per unit volume to molecular weight per unit volume. That is, the constants *C_B_* ≡ *N_B_* *ℓ_B_* *λ_B_* and *C_T_* ≡ *N_T_* *ℓ_T_λ_T_* may be regarded as the maximum velocities of resource conversion into mass. These products are expected to be quite large, in any case much larger than the other velocities at saturation such as *k_p_*, *k_v_*, *k_s_*, *k_m_* etc. We have written the diffusion term, ∇·[*D_Y_* ∇*_Y_*] in this generality to indicate that the diffusion factor *D_Y_* need not be constant.

If we prescribe the level of nutrients on the boundary of *D* and a given initial concentration of nutrients in *D*, with time, we expect the level of nutrients in the tissue to be low in regions “far” from the boundary. If the level of nutrient supply is sufficiently low, the tumor cells cannot proliferate except near the edge of the tumor. This is what is meant by “diffusion limited” tumor growth. This is the equation that controls the nutrient level in the tissue region, *D*.

Coupled to this should be an equation that reflects the diffusion of the products of cell metabolism in the tissue. Clearly the products of collagen degradation are among these products. Likewise, we should include the degradation products of p53, Sp1, MMP-1 and GF. However, since no further biochemical use of these waste materials is made in this article, we omit a detailed discussion of them.

## Boundary and Initial Conditions

We begin this section with a discussion of the boundary conditions. Since equations (2.1), (2.2), (2.6), (2.7), (2.11), (2.12), (2.13) are all ordinary differential equations in time, no boundary conditions are needed for them. On the other hand, (4.1), and (3.5) require boundary conditions. We take

(5.1) $$\begin{array}{r} D_{B}\eta_{B}n\cdot \nabla \;\text{ln}\;\left(\frac{\eta_{B}}{{𝔗}_{B}\;(m,f)\;\Psi_{B}\;(Z)}\right)=0,\\ D_{T}\eta_{T}n\;\cdot \;\nabla \;\text{ln}\;\left(\frac{\eta_{T}}{{𝔗}_{T}\;(m,f)\;\Psi_{T}\;(Z)}\right)=0,\\ \;\;D_{Y}n\;\cdot \;\nabla Y+r_{y}\;[Y\;(\text{x},t)-Y_{b}]=0 \end{array}$$

where *Z* is again the local volume fraction occupied by the cell types and where it is understood that **x** ε ∂ *D*, (the boundary of *D*) and where n denotes the outward directed unit normal to the boundary of *D*. The first two conditions say that the total flux of either cell type from the given region is zero. The flux constant *r_y_* needs to be determined from phenomenological considerations. The constant *Y_b_* is a prescribed level of nutrients on the outer wall.

We turn to a discussion of the initial conditions. Recall that a mutation is said to occur in a region *D'* ⊂ *D* over at time interval *I* if there has been a loss of p53 expression in the set *D'* × *I*. That is, equation (2.1) is replaced by (2.2). The function Ψ is called the loss of function coefficient and the strength of the loss of function is defined in (2.3). We start such a perturbed system with a stationary solution of the system of equations for which there are no tumor cells present and no loss of function. *Notice that we are not making any change in the initial values for either the proteins or the cell type densities. The only changes to be made are in the dynamics wherein the p*53 *equation takes the mutated form (2.2). The cell type equations are modified in that cell type change is driven only by the transfer rate* λ*_tr_*.

## Dynamics of the Modified Lotka-Volterra system

In order to understand what might be expected from the full system, we consider the set of stationary solutions of (3.5) in the spatially homogeneous case for constant values of *v*, *Y*, *f*, *m*. When the transfer coefficient λ*_tr_* = 0, we are led to consider a Lotka-Volterra competition model of the form:

(6.1) $$\begin{array}{l} \frac{dx}{dt}={\lambda^{\prime }}_{B}\;(x_{B}-x-L_{T}\;y)\;x,\\ \frac{dy}{dt}={\lambda^{\prime }}_{T}\;(y_{T}-y-L_{B}x)\;y, \end{array}$$

when we freeze the coefficients that depend on the switches and resources.

A complete discussion of the dynamical behavior of the solutions of this system to be found in [46]. One is tempted to believe that the solutions of the full system might behave like those for the system of ordinary differential equations. However there are two very good reasons why this may not be the case.

First, the presence of the free energy terms (i.e. the terms involving cell movement, both random and chemotactic or haptotactic), as well as the fact that the various parameters may depend upon the other variables, does not make such an expectation a rigorous statement. (As a well known example of how partial differential operators can radically alter the behavior of solutions of systems of ordinary differential equations, we refer the reader to a discussion of Turing instability [46].) See, for other examples, [11, 18, 21, 22, 19, 20, 34, 38] where studies have been made of related systems in which the Lotka-Volterra terms (the right hand sides in (6.1)) function as reaction terms in a system of reaction diffusion equations of competition type.

Secondly, the system of ordinary differential equations that corresponds to the spatially homogeneous cell equations can be reduced to the following form when we freeze the coefficients that depend on the switches:

(6.2) $$\begin{array}{l} \frac{dx}{dt}={\lambda^{\prime }}_{B}\;(x_{B}-x-L_{T}\;y)\;x-\Lambda \;x,\\ \frac{dy}{dt}={\lambda^{\prime }}_{T}\;(y_{T}-y-L_{B}x)\;y,+\Lambda \;x. \end{array}$$

This reduces to the Lotka-Volterra system when Λ = 0.

The “constants” *λ′*_*B*_, *λ′*_*T*_, *x_B_*, *y_T_* will depend upon *v*, *Y*, *μ_B_*, *μ_T_*, *λ_B_*, *λ_T_*, while Λ will depend upon λ*_tr_* and *p*. The three relevant ordinary equations from our model take the form:

$$\begin{array}{l} \;\;\;\;\;\;\;\;\;\;\;\frac{dp}{dt}\;=\;\frac{k_{p}Y}{K_{p}\;+\;Y}\;\frac{\eta_{B}}{K_{B}}\;-\;\mu_{p}p,\\ \frac{d\eta_{B}}{dt}\;=\;\left(\lambda_{B}^{\prime }\;H(v)\frac{y}{\Lambda_{B}\;+\;y}\left(1\;-\;\frac{\eta_{B}}{N_{B}}\;-\;L_{T}\;\frac{\eta_{T}}{N_{T}}\right)\;-\;\mu_{B}\right)\\ \;\;\;\;\;\;\;\;\;\;\;\;\;\;\eta_{B}\;-\;\lambda_{tr}H(p_{c}\;-\;p)\eta_{B},\\ \frac{d\eta_{T}}{dt}\;=\;\left(\lambda_{T}^{\prime }\;H(v)\;\frac{y}{\Lambda_{T}\;+\;y}\left(1\;-\;\frac{\eta_{T}}{N_{T}}\;-\;L_{B}\frac{\eta_{B}}{N_{B}}\right)\;-\;\mu_{T}\right)\\ \;\;\;\;\;\;\;\;\;\;\;\;\;\;\eta_{T}\;+\;\lambda_{tr}H(p_{c}\;-\;p)\eta_{B}. \end{array}$$

In order to understand how *p* affects the dynamics, we use smooth forms of the Heaviside function and consider the case of no loss of function, i.e. Ψ ≡ 0. To put this system into standard form, we freeze the values of *Y*, *v*, let *P_n_* be some normalizing constant for p53 and set

$$\begin{array}{l} \;\;\;\;\;\;\;x\;=\;\frac{\eta_{B}}{N_{B}},\;y\;=\;\frac{\eta_{T}}{N_{T}},\;z\;=\;p/P_{n},\\ \lambda_{1}\;=\;\lambda_{B}H(v),\;K_{bv}\;\frac{Y}{\Lambda_{B}\;+\;Y},\;\mu_{1}\;=\;\mu_{B},\\ \;\;\;\;\;\;\;\;\;\;k_{1}\;=\;L_{T},\;x_{0}\;=\;1\;-\;\mu_{1}/\lambda_{1},\\ \lambda_{2}\;=\;\lambda_{T}H(v),\;K_{tv}\;\frac{Y}{\Lambda_{T}\;+\;Y},\;\mu_{2}\;=\;\mu_{T},\\ \;\;\;\;\;\;\;\;\;\;k_{2}\;=\;L_{B},\;y_{0}\;=\;1\;-\;\mu_{2}/\lambda_{2},\\ \lambda_{3}\;=\;\frac{(k_{p}/P_{n})Y}{K_{P}\;+\;Y},\;\mu_{3}\;=\;\mu_{p},\;g(z)=\;\lambda_{tr}h(z) \end{array}$$

where *h*(*z*) is a smooth approximation of *H*(*p_c_*/*P_n_* −*z*). In terms of these quantities the system becomes

(6.3) $$\begin{array}{l} x^{\prime }=F\;(x,y,z)=\lambda_{1}x\;(x_{0}-x-k_{1}y)-xg\;(z)\\ y^{\prime }=G\;(x,y,z)=\lambda_{2}y\;(y_{0}-y-k_{2}x)-xg\;(z)\\ z^{\prime }=H\;(x,y,z)=\lambda_{3}x-\mu_{3}z \end{array}$$

If the transfer rate *λ_tr_* = 0, so that *g* = 0, then the first two equations decouple from the third equation, yielding a planar system for *x* and *y* which is a Lotka-Volterra competition model with four rest points: i) (0, 0), ii) (*x*_0_, 0), iii) (0, *y*_0_) and iv) (*x*_1_, *y*_1_), the point of intersection between the lines *x*_0_ − *x* − *k*_1_*y* = 0 and *y*_0_ − *y* − *k*_2_*x* = 0. The point (*x*_1_, *y*_1_) is not always physically relevant. Depending on *k*_1_, *k*_2_ it may not be in the first octant, or it may not satisfy *x*_1_ + *y*_1_ ≤ 1. The dynamics of the system also varies with *k*_1_, *k*_2_. A thorough discussion can be found in [46]. In the case where the parameters are such that *x*_0_ = *y*_0_, *k*_1_ = *k*_2_ and *k*_1_*k*_2_ > 1, The points (*x*_0_, 0) and (0, *y*_0_) are asymptotically stable. These two, as well as a third, (*x*_1_, *y*_1_) are physically relevant (i.e. *x*_1_ > 0, *y*_1_ > 0, *x*_1_ + *y*_1_ ≤ 1). However, (*x*_1_, *y*_1_) is unstable.

**Figure 2.**
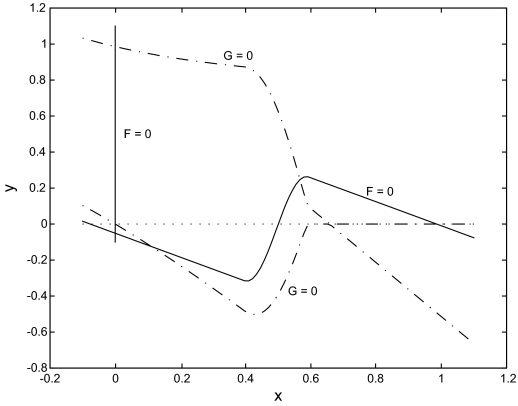
The null clines of the reduced planar system F(*x*, *y*, *α x*) = 0 (solid curves) and G(*x*, *y*, *αx*) = 0 (dashed curves). The rest points are the intersection points of a solid curve with a dashed curve. Notice that there is one with a negative y coordinate.

The rest points of the full 3-dimensional system (6.3) satisfy *F* = *G* = *H* = 0. Since *H* = 0 if and only if *z* = *αx*, where *α* = *λ*_3_/*μ*_3_, the 3-d system can be reduced to a planar system for *x* and *y*; namely *F* (*x*, *y*, *αx*) = *G*(*x*, *y*, *αx*) = 0. This system can be analyzed graphically by plotting the curves *F* (*x*, *y*, *αx*) = 0, *G*(*x*, *y*, *αx*) = 0 and looking for points of intersection. *For the choice of parameters and smooth approximation to the Heaviside function used in the simulations*, *this approach leads to* Figure 2.

Figure 2 shows there are now 5 rest points, one of which is not phyically relevant. The solutions can be found numerically and a linear stability analysis can also be performed numerically. Table 2 gives the results of this process for the physically relevant rest points. *These results show that for the reduced system (6.3) with smooth approximations to the Heaviside function, there can be an equilibrium state with co-existing benign and tumor cell densities that is unstable!*

## Steady state solutions

Before we can perform simulations to explore the effects of modeling mutational loss of function in p53, steady states of the system must be determined. Since we cannot expect spatially homogeneous steady states, equilibrium solutions must be determined computationally, rather than algebraically. To do this, we use a shooting strategy which relies on asymptotic stability. That is, we choose initial conditions for the system and then let the system evolve. If the initial conditions are nearby an asymptotically stable steady state, then the evolving time-dependent solution will converge to the nearby equilibrium solution. This raises the question of how to choose appropriate initial conditions, which we discussed in the first subsection. Essentially we use constant solutions of a perturbed system. These values also serve as normalizing values for some of the variables. A second question arises along the way, that of an appropriate boundary value *Y_b_*. This is discussed in the second subsection. Simulation results are also presented.

### Initial conditions and normalizing constants

In this subsection we treat *Y_e_* as a parameter and seek spatially constant solutions of (2.1), (2.6), (2.7), (2.11), (2.12), (2.13), (3.5) and the following perturbation of (4.1):

(7.1) $$\begin{array}{r} \frac{\partial Y}{\partial t}=\nabla \cdot [D_{Y}\nabla Y]+Y_{r}-Y\;(\text{x},t)\\ \;\;𝔖\;(Y,f,s_{B}^{a},s_{T},v,\eta_{B},\eta_{T}) \end{array}$$

where 𝔖 is defined implicitly in (4.1) and where *Y_r_* is to be chosen so that there is a constant stationary solution for the system (2.1), (2.6), (2.7), (2.11), (2.12), (2.13), (7.1), and (3.5).

To determine this solution, we set the right hand sides of (2.1), (2.6), (2.7), (2.11), (2.12), (2.13), (3.5) to zero, assume that the variables in the resulting equations are constants, set all the Heaviside switches to unity except that we set *H*(*p_c_* − *p*) = 0 in order to indicate that there is no transfer of cells from the benign state to the malignant state at *p* = *p_e_* > *p_c_* where *p_e_* is an equilibrium value to be determined as explained below.

We use the underlying assumption that $\eta_{B}^{e}$ > 0 and $\eta_{T}^{e}$ =0. Therefore $s_{T}^{e}$ = 0. For the benign variable we obtain a normalized solution, $\eta_{B}^{e}\;/\;N_{B}$, as

(7.2) $$N_{B}^{e}=\frac{\eta_{B}^{e}}{N_{B}}=1-\frac{\mu_{B}}{Y_{B}^{e}},\left(Y_{B}^{e}=\frac{\lambda_{B}Y_{e}}{K_{B}+Y_{e}}\right).$$

In order to have $N_{B}^{e}$ > 0 we need *μ_B_* < $Y_{B}^{e}$ This is an inequality in *Y_e_* that is equivalent to *Y_e_* > *μ_B_* *λ;_B_/*(λ*_B_* – *μ_B_*) ≡ Y_B_) It is also clear that $N_{B}^{e}$ → 0^+^, as *Y_e_* → $Y_{B}^{+}$.

From the remaining equations we obtain:

(7.3) $$\begin{array}{ll} & p_{e}=\frac{Y_{p}^{e}N_{B}^{e}}{\mu_{p}},\left(Y_{p}^{e}=\frac{k_{p}Y_{e}}{K_{p}+Y}\right),\\ s_{e}\equiv & s_{B}^{e}=\frac{Y_{s}^{e}N_{B}^{e}}{\mu_{sB}},\left(Y_{s}^{e}=\frac{k_{s}Y_{e}}{K_{s}+Y_{e}}\right),\\ & s_{e}^{a}=\frac{s_{e}}{1+\nu_{e}^{s}p_{e}}. \end{array}$$

Let

$$Y_{v}^{e}\;=\;\frac{k_{v}Y_{e}}{K_{v}\;+\;Y_{e}}\;\text{and}\;Y_{r}^{e}\;=\;\frac{K_{\text{cat}}^{r}Y_{e}}{K_{m}^{r}\;+\;Y_{e}}.$$

Then for *v_e_* and *m_e_*, it follows that

(7.4) $$v_{e}=\frac{Y_{v}^{e}N_{B}^{e}}{\mu_{v}+Y_{r}^{e}N_{B}^{e}},\;\text{and}\;m_{e}-\frac{\eta_{m}v_{e}Y_{r}^{e}N_{B}^{e}}{\mu_{m}}.$$

To determine a constant solution *f_e_* > 0 of (2.13), we set *F*_*e*_ = *f*_*e*_/*f*_*M*_, ${\tilde{K}}_{cat}^{f}=K_{cat}^{f}\;m_{e}\;/\;f_{M}$, and ${\tilde{K}}_{m}^{f}=K_{m}^{f}\;/\;f_{M}$. Depending on parameter values there may or may not be a positive solution of the stationary equation for (2.13). If we set *x = F_e_*, $\alpha =Y_{f}^{e}N_{B}^{e}$, $\beta ={\tilde{K}}_{cat}^{f}$, and $\gamma ={\tilde{K}}_{m}^{f}$. It can be shown that if γ ≥ 1, there will be a positive solution if and only γ > *β/α*. It turns out that the stationary equation will have distinct real zeros if and only if *β/α* < (γ + 1)^2^/4. In this case, the larger one is given by $x=\frac{1}{2}(1-\gamma +\sqrt{{\left(\gamma +1\right)}^{2}-4(\beta /\alpha )})$. If γ < l this root is positive. Since γ ≤ (γ + 1)^2^/4, it follows that the weaker requirement *β/α* < (γ + 1)^2^/4 is necessary and sufficient for *x* > 0 when γ<1. If the constant *Y_r_* is now determined by setting *Y_r_* *= Ye*𝔖*(Y_e_, f_e_, s_e_, 0, v_e_,* $\eta_{B}^{e}$,0), then the above values give the desired constant solutions of (2.1), (2.6), (2.7), (2.11), (2.12), (2.13), (7.1), (3.5).

**Remark 2.** Notice that *P_e_*, …, $\eta_{B}^{e}$ depend only upon the kinetic constants, the proliferation and apoptosis rates and *Y_e_*, the level of resources.

### The boundary value *Y_b_*

The constant values found above are not steady states of the original system in which (4.1) appears rather than (7.1). In fact constant solutions are not possible. Clearly the distribution of resources cannot be spatially homogeneous, since *Y_s_* = *Y*𝔖 (*Y*, *f*, *v*, $s_{B}^{a}$, *s_T_*, *η_B_, η* *_T_*) > 0, unless all variables are zero. However, there are spatially inhomogeneous equilibrium solutions of the system that are close to these constant values, provided that the flux of nutrients at the boundary diffuses through the region resulting in a distribution of resources approximating the distribution determined by *Y_r_*. The key issue is to determine the proper boundary value for *Y_b_*. This can be done analytically as follows.

Suppose that *Y*_0_ (*x*) is the resource component of an equilibrium solution of the model system (with *Y_r_* = 0) in which there are no tumor cells present. Then *Y*_0_ (*x*) satisfies

(7.1) $$\begin{array}{l} 0=\nabla \cdot (D_{Y}\nabla Y)-Y_{s}\;(Y,f,v,s_{B,}^{a}0,\eta_{B},0),x\in D,\\ 0=D_{Y}\frac{\partial Y}{\partial v}+r_{y}\;(Y-Y_{b}),x\in \partial D, \end{array}$$

where *D* is the region of interest, $\frac{\partial Y}{\partial v}$ denotes the outward normal derivative and $s_{B}^{a}$, *v*, *f*, *Y*, *η_B_* are the corresponding steady states of the full system. If *Y*_0_*(x)* ≈ *Y_e_* throughout *D* then we would expect their average values to be approximately the same. We use this observation to determine *Y_b_*, and hence the flux at the boundary, by requiring

$$\frac{1}{\left|D\right|}\int_{D}Y_{0}(x)dx\;=\;Y_{e},\;\;\;\left(|D|\;=\;\int_{D}1\;dx.\right)$$

Suppose that &*Ycirc;* (*x*) is the solution of (7.1) satisfying the homogeneous boundary condition $D_{Y}\frac{\partial Y}{\partial v}+r_{y}Y=0$, *x* ε ∂*D*. Since the equations (7.1) are linear and *Y_b_* is constant, the principle of superposition yields *Y*_0_ (*x*) *= Ŷ (x) + Y_b_*. Therefore $Y_{b}=\frac{1}{\left|D\right|}\int_{D}\;(Y_{0}\;(x)-\hat{Y}\;(x))\;dx=Y_{e}-\frac{1}{\left|D\right|}\int_{D}\hat{Y}\;(x)\;dx$. Thus, to determine *Y_b_*, it is sufficient to determine the mean value of *Ŷ*(*x*). If the values of $s_{B}^{a}$, *v*, *f*, *Y*, *η_B_* are approximately the same as the normalizing values *s_e_*, *v_e_*, *f_e_*, *Y_e_*, $\eta_{B}^{e}$ then we should have *Ŷ* (*x*) ≈ *Ŷ_e_*(*x*), where *Ŷ*(*x*) satisfies 0 = ∇·(*D_Y_* ∇ *Y*) − $Y_{s}^{e}$, *x* ε *D*, $D_{Y}\frac{\partial Y}{\partial V}+r_{y}Y=0$, *x* ε ∂*D,* where $Y_{s}^{e}=Y_{e}\;𝔖(Y_{e},f_{e},s_{e},0,v_{e},\eta_{B}^{e},0)$ is a constant. Clearly $Y_{s}^{e}=Y_{r}$ by the choice of *Y_r_* that was made to obtain the normalizing constants.

In the case that $D=\left\{x\in R^{3}:\left\|x\right\|<R\right\}$ is the ball of radius *R* centered at the origin, then *Ŷ_e_*(*x*) can be explicitly computed to be ${\hat{Y}}_{e}(r)=-\frac{r_{s}^{e}}{3}(\frac{R}{r_{y}}+\frac{R^{2}-r^{2}}{2D_{r}})$. Integration yields $\overset{\int }{D}{\hat{Y}}_{e}(x)dx=-\frac{Y_{s}^{e}}{3}(\frac{R}{r_{y}}\frac{4\pi R^{3}}{3}+\frac{4\pi R^{5}}{15D_{Y}})$, and hence $Y_{b}=Y_{e}+\frac{Y_{s}^{e}}{3}(\frac{R}{r_{y}}+\frac{R^{2}}{5D_{Y}})$ as an appropriate resource level at the boundary.

### Evolution to Equilibrium

All our computations were done with the system in non-dimensionalized form. The non-dimensionalization of the variables is given in Table 3. To computationally determine steady states we used a shooting strategy. We were able to determine an asymptotically stable steady state of the system, as predicted by the reduced system, that has a nearly constant benign cell population with no tumor cells present. It was possible to use the constant states found in section 7.1 as initial values and successfully evolve to this steady state. In terms of the non-dimensional variables many of the initial values were equal to one. However, a set of initial values that lies closer to this steady state is obtained if the values of *p*, *η_B_* and *η_T_* are chosen instead to be the (constant) solutions of the reduced system, with *v = v_e_, Y = Y_e_* fixed that are given in Table 5.

**Figure 3.**
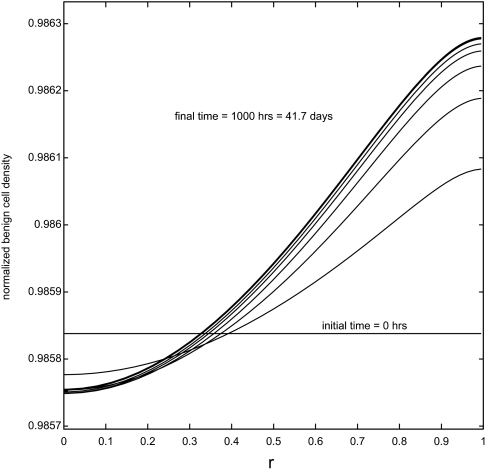
Spatial profiles of *N_B_* (*r*, *t*) with *t* = *t_i_* = *i*Δ*t*, where Δ*t* = 100 hours. As time increases the profiles monotonically approach an equilibrium solution profile.

**Figure 4.**
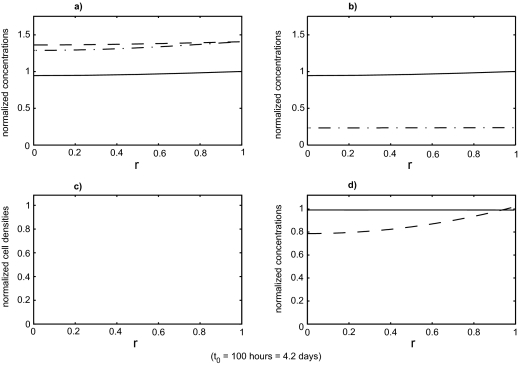
The state of the system just prior to the start of the loss of function event. All variables have been non-dimensionalized. The system was initialized to its equilibrium configuration. During the first 100 hours of the simulation it remains in an equilibrium state which is shown at the end of this 100 hour period. The loss of function event was then initiated. The functions plotted are: a) growth factor, *v* (– –), protease, *m* (– ^.^), p53, *p* (—); b) total benign Sp1, *s_B_* (—), active benign Sp1, $S_{B}^{a}$ (– .), tumor Sp1, *s_T_* (– –); c) benign cell density, *η_B_* (—), tumor cell density, *η_T_* (– –); d) fibronectin, *f* (—), resources, *Y* (– –).

Figure 3 shows the evolution of the benign cell density, starting with the initial values of *s_B_*, *s_T_*, *v*, *m*, *f* and *Y* chosen as the constants from section 7.1 and the initial values of *p*, *η_B_* and *η_T_* chosen as the solutions of the reduced system (with all values non-dimensionalized). Spatial profiles at increments of 100 hours are superimposed in this plot. After a fairly rapid initial transient the profiles gradually approach an equilibrium. All other variables in the system were observed to approach their equilibrium profiles much more rapidly than the benign cell density. The equilibrium states of the full system are shown in Figure 4.

## Results and Conclusions

It is clear that the system of equations and boundary conditions is quite involved. It is perhaps worth taking a paragraph or two to explain what one might expect from the replacement of (2.1) by (2.2). The implementation of the loss of function is through equation (2.2) and is controlled by the loss of function coefficient. Suppose that the level of p53 falls below the critical level. Then there will be a small gain in tumor cell density. This will drive, through the equation for tumor induced Sp1, an increase in growth factor production and hence an increase in MMP-1 production as one sees from the first term in (2.12). This in turn leads to a degradation of matrix collagen/fibronectin as is indicated by the sink term in (2.13). If there are no tumor cells initially present, this small gain in tumor cell density will drive the production of tumor expressed Sp1. The tumor expressed S*p*1 is not under the regulation of p53 and hence can induce an increase in the rate of growth factor production. The induced concentration gradients in these two proteins in turn stimulate the chemotactic movement of the two cell types.

As presented in (2.2), the loss of function is modeled by the expression 1 – Ψ(*x, t*). Suppose that Ψ(*x*, *t*) = I φ (x)Ψ(t) where: i) φ(*x*) = 1 for *x ε D'* and is zero elsewhere, ii) *Ψ*(*t*) = 1 for *t*_0_ *< t < t*_0_ *+ T* and is zero elsewhere (for some *t*_0_), and iii) I∈[0,1] is an amplitude factor. Then *T* is the duration of the loss of function event, and I is the intensity of the loss of function, with I=0 corresponding to no loss of function and I=1 being complete loss of function. The domain *D*′ can also be manipulated, but we will focus more on duration and intensity. For example, suppose *D*′ *= B*(*r*_0_) is the ball about zero of a fixed radius *r*_0_ < *R*. Then *S* = $4T\pi r_{0}^{3}T/3$ is the strength of the loss of function. When S=0=I there is no loss of function while when $S=4T\pi r_{0}^{3}\;/\;3$, the loss of function is complete on *B*(*r*_0_) × [*t*_0_, *t*_0_ + *T*].

The simulations were done using a spherical domain *D*, with the origin of the coordinate system at the point where loss of function occurs. Radial symmetry was assumed, so that all functions depended only on *r = |x|*, the distance to the origin. The domain was non-dimensionalized to the ball bounded by the sphere of radius *R =* 1. The domain *D*′ was taken to be a ball with a smaller radius. As with the other step functions appearing in the model description, smoothed versions of the functions φ(*x*) and *ψ*(*t*) defining Ψ(*x*, *t*) were used in the 1 simulations. In particular. we chose $\varphi (r)=\frac{1}{2}(1+\text{cos}((r-r_{0}+\delta )\pi /(2\delta ))$, for *|r – r*_0_| ≤ *δ*, with φ(*r*) = 1 to the left of this interval and φ(*r*) = 0 to the right. Thus, *D*′ was a ball of radius *r*_0_ + *δ*.

### Basic Simulations

The model will demonstrate that the loss of p53 function can lead to the uncontrolled growth of a malignant avascular tumor if the loss is sufficiently strong, occurs for a sufficiently long time or involves a sufficiently large number of cells (as measured by the product of cell density times volume). On the other hand if any one of these three factors is sufficiently small, regardless of the intensity of the others, the loss of function will be harmless in the sense that any initial tumor formation will decay. The loss of function is manifested in the model by a decline in the mitosis rate for benign cells together with an increase in the mitosis rate for tumor cells, both **rates** having the same (maximum) magnitude *λ_tr_η_B_* where *η_B_* is the local density of benign cells.

In particular, the model demonstrates the existence of a critical intensity, $I_{c}$ such that if the intensity is below this number, any tumor formed during the time period during which loss of function is experienced will decay once the loss of function is no longer being experienced. On the other hand, if the intensity is larger than this critical intensity, then there is a critical time duration, $T_{I}$, such that if the loss of function is experienced for any finite time longer than this critical time, the formed tumor will grow and take over the entire region. (Recall that this region is only one or two *mm* in diameter and that we are dealing only with solid tumor growth.) If the loss of function occurs only for a time duration smaller than this critical time, the solid tumor will eventually decay.

*In this case, the model suggests the existence of an unstable tumor cell region coexisting with the benign cell region which will form if the loss of function is experienced for the critical duration*. From a practical point of view, one can view the formation of such a tumor cell region as occurring if the duration time is only close enough to the critical time. For example if the doubling time (when T > $T_{I}$) or half life (when (T < $T_{I}$) is much larger than the remaining life expectancy of the individual, we can view the tumor to be in a quasi steady state with its surroundings.

**Figure 5.**
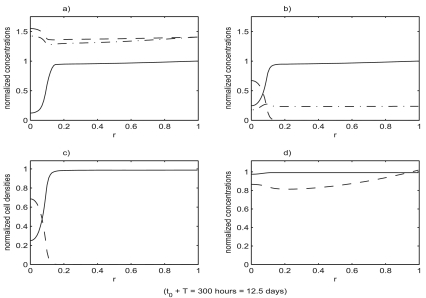
The state of the system just after the end of the loss of function event. Functions plotted are the same as in Figure 4. The intensity of the event in the simulation was I=0.5, that is a 50% loss of function. The duration of the event was *T* = 200 hours. The state of the system at the end of the event is shown. For subsequent times the system evolves with no loss of function.

**Figure 6.**
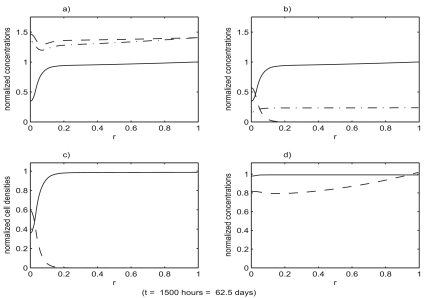
The state of the system in Figure 5 at a later time. Functions plotted are the same as in Figure 4. The system has continued to evolve and eventually returns to the original equilibrium state. An immediate time between the end of the loss of function event and full recovery is shown.

**Figure 7.**
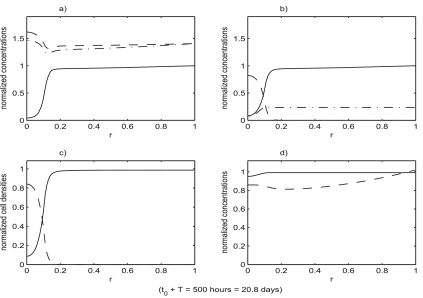
The state of the system just after the end of the loss of function event. Functions plotted are the same as in Figure 4. The intensity of the event in the simulation was I=0.5, that is a 50% loss of function. The duration of the event was *T* = 400 hours. The state of the system at the end of the event is shown. For subsequent times the system evolves with no loss of function.

**Figure 8.**
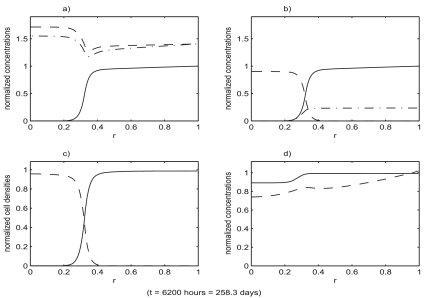
The state of the system in Figure 7 as the tumor cell population expands, causing the density of benign cells to fall dramatically in the region surrounding the location where the loss of function occurred. The spread of the tumor cell population and the decline of benign cell population progresses in the form of a traveling wave. Functions plotted are the same as in Figure 4.

**Figure 9.**
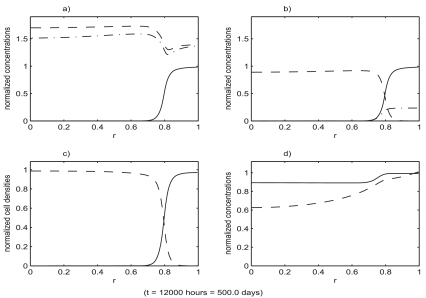
The state of the system shown in Figures 7, 8 at a much later time. Functions plotted are the same as in Figure 4. The tumor cell population has almost completely displaced the benign cell population in the entire region.

Figures 4–6 show the result of a loss of function event that does not result in tumor growth. Figures 7–9 show the result of a loss of function event that does result in tumor growth. Both simulations were started with the system in equilibrium and allowed to evolve, maintaining equilibrium, for 100 hours. The equilibrium state is shown in Figure 4. After a very brief one hour transition the loss of function event begins. The intensity of the event is the same in both cases, I=0.5; that is a 50% loss of function. The parameters determining φ(*r*) were also the same in both cases: *r*_0_ = 0.1, *δ* = 0.05. After *T* hours, another brief one hour transition to a system with no loss of function is made. These times are recorded in the figures, with the one hour transitions ignored. The durations were *T* = 200 hours in the first case and *T* = 400 hours in the second case.

### Threshold and Critical Values

Given a level of intensity I for a loss of function event, there may be a critical value $T_{I}$ of the time of duration such that if $T>T_{I}$ then the event results in tumor growth, while if $T>T_{I}$ then the event does not result in tumor growth. The simulation results presented in the previous subsection clearly suggest this is the case when I=0.05. It is also intuitively clear that if the intensity is extremely small then no significant tumor growth will occur, regardless of the duration of the loss of function event. In other words, there should be a threshold of intensity that must be exceeded before significant tumor growth can occur after a sufficiently long loss of function event.

The roles of I and *T* can be reversed and one can ask, for a given time of duration *T*, whether or not there is a critical intensity $I_{c}$. From this point on view, one would expect a threshold for the time of duration that must be exceeded for significant tumor growth to occur even when the intensity is as large as possible.

Threshold values for both I and *T* are predicted by the model. In the model, a loss of function event results in a decrease in the production of p53. If the amount being produced does not fall below the critical value *p_c_* then the transfer term, λ*_tr_* *H*(*p_c_* *– p*)*η_B_*, appearing in the cell equations (3.5) will remain zero and no change in the dynamics should be expected. (However, there will be a subtle change in the growth factor and resource level that can change production levels slightly.) In order to have the level of p53 fall below the critical value, the loss of function event must have a sufficient intensity, and this results in thresholds for both I and *T*.

**Figure 10.**
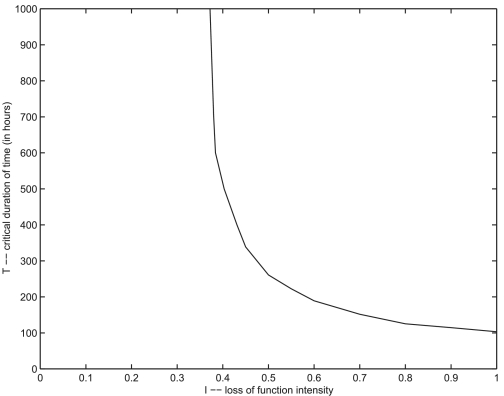
The curve of critical points that indicates threshold values for tumor growth.

The graph in Figure 10 shows the functional dependence of the critical time $T_{I}$ of duration on the intensity for loss of function events as predicted by the model. It also shows the threshold value for *T* at maximum intensity I=1. The threshold for I is indicated by the vertical asymptote. The curve also depends on the size of the region *D*′ where the loss of function occurs. For the plotted curve, the same function φ(*r*) described above was used, with the parameters determining φ (*r*) being *r*_0_ = 0.10, λ = 0.05. To determine points on the curve, a bisection technique was used. For example, for a given value of I, two bracketing values *T*_0_ *< T*_1_ of the time of duration were found such that the system returned to equilibrium if the time of duration was *T*_0_, or experience overwhelming tumor growth if the time of duration was *T*_1_. Then the average of these times *T_m_* *= (T*_0_ + *T*_1_ *)/*2 was used as the duration for the loss of function event in a simulation experiment. If the system returned to equilibrium then *T*_0_ was replaced by *T_m_*, otherwise *T*_1_ was replaced by *T_m_*. Then the experiment was repeated.

**Figure 11.**
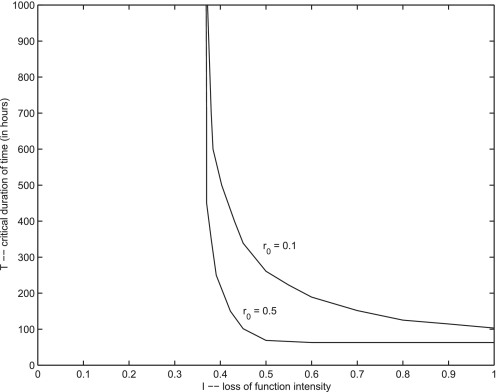
Curves of critical points that indicates threshold values for tumor growth. The lower curve is corresponds to a domain *D*′ and function φ(*r*) determined by the parameters *r*_0_ = 0.5, δ = 0.05. For the upper curve *r*_0_ = 0.10, δ = 0.05.

When the domain *D*′ over which the loss of function takes place increases in size the intensity or duration of the event is diminished. In Figure 11 two curves of critical points are plotted. The lower curve was determined using a domain *D*′ and function *φ*(*r*) determine by the parameters *r*_0_ = 0.5, *δ* = 0.05, while the upper curve corresponds to *r*_0_ = 0.10, *δ* = 0.05 as in Figure 10. Because mutations in one location are very rare, the domain of a single the loss of function event is expected to have roughly the volume of a single cell or two.

## Figures and Tables

**Table 1.** **Notation.**The standard chemist’s notation for the concentration of a species *X* is [*X*]. Using such a notation for functions which depend on spatial as well as temporal variables, especially in combination with sub and superscripts, is clumsy. To simplify this, we use the notation in the table below. Furthermore, if a molecular species is associated with a benign cell or with a tumor cell we use the subscripts *B*, *T* with its label. For example, *s_B_*, *s_T_* refer to the concentrations of Sp1 in the benign and in the tumor cells respectively.

Species	Notation	Function form	Equation number
p53 protein	*P*	*p*(**x**,*t*)	(2.1)
Sp1, benign cell derived GF transcription factor	*T_r_*	*S_B_*(**x**,*t*)	(2.6)
Sp1, tumor cell derived GF transcription factor	*T_r_*	*S_T_*(**x**,*t*)	(2.7)
Sp1 in activated state	$T_{r}^{a}$	$S_{B}^{a}\;(\text{x},t)$	(2.8)
Sp1 inhibited by p53	$T_{r}^{i}$	$S_{B}^{i}\;(\text{x},t)$	(2.8)
GF, growth factor	*V*	*v*(**x**,*t*)	(2.11)
MMP-1, matrix metalloproteinase-1	*M*	*m*(**x**,*t*)	(2.12)
Tissue collagen/fibronectin proteins	*F*	*f*(**x**,*t*)	(2.13)
nutrients (amino acids, lipids, oxygen, sugars, etc.)	*Y*	*Y*(**x**,*t*)	(4.1)
benign cell density	*B*	*η_B_*(**x**,*t*)	(3.5)
malignant cell density	*T_m_*	*η_T_*(**x**,*t*)	(3.5)

**Table 2.** Numerically determined rest points of physical relevance (rounded to 3 decimal places) for the system (6.3) and their stability properties.

X	y	z	Dynamics
0.000	0.000	0.000	unstable, 1-D unstable manifold
0.000	0.986	0.000	asymptotically stable
0.567	0.247	0.573	unstable, 1-D unstable manifold
0.986	0.000	0.996	asymptotically stable

**Table 3.** **Nondimensionalized variables.**Except for length and time scales, the normalizing constants in this table were computed in subsection 7.1.

Quantity	Variable	Dimensionless variable
time	*t*	*τ* = *t/*t
position	**x**	𝔵 = **x***/L*
p53	*p*(**x**, *t*)	*P*(𝔵,*τ*) *=p*(**x**, *t*)*/p_e_*
Sp1	*s_B_*(**x**, *t*)	S*_B_* (𝔵,*τ*) = s*_B_* (**x**, *t*)/*s_e_*
active Sp1	$S_{B}^{a}\;(\text{x},t)$	$S_{B}^{a}\;(𝔵,\tau )=s_{B}^{a}\;(\text{x},t)/s_{e}$
Sp1	s*_T_*(**x**, *t*)	S*_T_* (𝔵,*τ*) = *s_T_* (**x**, *t*)/*s_e_*
MMP-1	*m*(**x**, *t*)	M(𝔵,*τ*) = *m*(**x**, *t*)/*m_e_*
GF	*v*(**x**, *t*)	V(𝔵,*τ*) = *v*(**x**, *t*)/*v_e_*
collagen	*f*(**x**, *t*)	*F*(𝔵*,τ*) *= f*(**x**, *t)/f_M_*, *F_e_= f_e_/f_M_*
Nutrients	*Y*(**x**, *t*)	*Y*(𝔵,*τ*) *= Y(***x**, *t*)*/Y_e_*
benign cell fraction	*η_B_*(**x**, *t*)	*N_B_* (𝔵,*τ*) *=η_B_(***x**, *t*)*/N_B_*
tumor cell fraction	*η_N_*(**x***, t*)	*N_T_* (𝔵,*τ*) *= η_T_* (**x**, *t*)*/N_T_*
cell fraction total	*η*(**x***, t*) *= η_B_*(**x***, t*)*/N_B_+ η_T_*(**x***, t*)*/N_T_*	N(𝔵,*τ*) *=* N*_B_* (𝔵,*τ*) *+* N*_T_* (𝔵,*τ*)

**Table 4.** Numerical values used in simulations.

Equation	Constants	Values	References
	*R*	0.100(10^1^) mm	simulated value
(2.1)	*k_p_*	0.114(10^0^) h^−1^	see notes
	*K_p_*	0.500(10^3^) *μ*M	simulated value
	*μ_P_*	0.140(10^1^) h^−1^	see notes
(2.6),(2.7)	*k_s_*	0.644(10^−1^) h^−1^	see notes
	*K_s_*	0.500(10^3^) *μ*M	simulated value
	*μ*_*S*_*B*__	0.180(10^1^) h^−1^	see notes
	*μ*_*S*_*T*__	0.180(10^1^) h^−1^	see notes
(2.8)	*V_e_*	0.500(10^2^) (*μ*M) ^−1^	simulated value
(2.11)	*k_v_*	0.313(10^0^) h^−1^	see notes
	*K_v_*	0.500(10^3^) *μ*M	simulated value
	*μ_v_*	0.208(10^1^) h^−1^	see notes
	$K_{cat}^{r}$	0.300(10^1^) h^−1^	simulated value
	$K_{m}^{\text{r}}$	0.500(10^3^) *μ*M	simulated value
(2.12)	*μ_M_*	0.416(10^1^) h^−1^	see notes
	*n_m_*	0.800(10^1^)	simulated value
(2.13)	*T_f_*	0.180(10^2^) h	see notes
	*K_f_*	0.500(10^3^) *μ*M	simulated value
	*f_M_*	0.140(10^4^) *μ*M	see notes
	*ε_f_*	0.100(10^0^)	simulated value
	$K_{cat}^{f}$	0.170(10^2^) h^−1^	simulated value
	$K_{m}^{f}$	0.700(10^3^) *μ*M	simulated value
(3.5)	*N_B_*	0.100(10^13^) l^−1^	see notes
	*D_B_*	0.360(10^−5^) mm^2^h^−1^	see notes
	*λ_B_*	0.120(10^−1^) h^−1^	see notes
	*K_B_*	0.500(10^3^) *μ*M	simulated value
	*L_T_*	0.150(10^1^)	simulated value
	*μ_B_*	0.100(10^−3^) h^−1^	see notes
	*λ_tr_*	0.750(10^−2^) h^−1^	see notes
	*N_T_*	0.100(10^13^) l^−1^	see notes
	*D_T_*	0.360(10^−5^) mm^2^h^−1^	see notes
	*λ_T_*	0.120(10^−1^) h^−1^	see notes
	*κ_T_*	0.500(10^3^) *μ*M	simulated value
	*L_B_*	0.150(10^1^)	simulated value
	*μ_T_*	0.100(10^−3^) h^−1^	see notes
(4.1)	*D_Y_*	0.500(10^−5^) mm^2^h^−1^	simulated value
	*ℓB*	0.200(10^−5^*) μ*M	simulated value
	*ℓT*	0.200(10^−5^)	simulated value
(5.1)	*r_y_*	0.100(10^1^) mmh^−1^	simulated value

**Table 5.** **Derived normalizing constants used in simulations.**The choice of Y*_e_* given in the table determines normalized benign cell density to be $\eta_{B}^{e}$/*N*_*B*_ = 0.990, *f_e_* = 0.139(10**^4^**)*μ*M. and the normalizing constants given in the table.

variable	value
*Y_e_*	0.200(10^4^) *μ*M
*p_e_*	0.645(10^−1^) *μ*M
*s*_e_	0.283(10^−1^) *μ*M
*v_e_*	0.278(10^−1^) *μ*M
*m_e_*	0.127(10^0^) *μ*M

## Footnotes

1 The Heaviside function is only defined “almost everywhere”. Typically, one splits the difference and sets *H*(0) = 1/2.

2 The chemical mechanism for collagen degradation is *M + F* ⇄ {MF} → *M + F′* where *F′* denotes the products of matrix degradation. The Michealis-Menten hypothesis yields the second term in (2.13). The logistic type term *f* (1–*f*/*f**_M_*) must be modified to reflect cellular expression of matrix protein and hence the inclusion of the normalized cell concentrations. This can be made rigorous using kinetics but limitations of length preclude us doing so here.

## Appendix A. Biochemistry

In this section, using a kinetic approach to the molecular events involved in transcription and translation, we derive equations (2.1), (2.6), (2.7), (2.11), and (2.12). Because the actual events are quite complex, we take an overall approach to them. Our purpose is to give some justification for the simple relationship used in these equations from a kinetic point of view.

The sources of the nucleotides and amino acids involved in the assembly of the mRNA and proteins must ultimately be the blood and tissue.

For our purposes, a cell consists of two regions, the nucleus and the cytoplasm separated by a membrane. The transcription of a gene into RNA and its maturation into mRNA takes place in the former. The mRNA is transferred from the nucleus across the nuclear membrane into the cytoplasm. There the ribosome translates it, via the genetic code, into a protein. This protein may stay associated with the cell or it may be secreted into the extra cellular matrix (ECM) where it may perform several functions. For example, it might be a protease that degrades the ECM or a growth factor that binds to a receptor on the same or another cell (that need not be of the same type as the parent cell) to initiate the expression of other genes via some signal transduction pathway.

Clearly, the sequence of events involved in transcription and translation will involve biochemical mechanisms and processes that may be only incompletely understood quantitatively as well as qualitatively. In order to obtain the “starting” material from the ECM and plasma, one has to transfer these molecules across the cell membrane and, in the case of the nucleotides, across the nuclear membrane. This is typically accomplished by diffusion. There are transmembrane proteins that permit the transfer of such small molecules.

We take the point of view in all of this that the rate limiting step is the translation step in the cytoplasm: That is, the ribosome (Rb), the cytoplasmic *mRNA* (*^c^**Rn*) and the *tRNA* (not shown) work to assemble the protein from the amino acids in the cytoplasm:

(A.1) $$\begin{array}{l} {}^{c}Rn+Rb\overset{k_{on}}{\underset{k_{off}}{⇄}}{}^{c}RnRb,\\ Y+{}^{c}\;RnRb\overset{k}{\to }Rb+{}^{c}N+n\;{}^{c}P_{G} \end{array}$$

(Here *n* represents the number of molecules of the protein that are assembled during the life time of the *mRNA*.)

However, if we assume that the second of equations (A.1) is rate limiting and we apply the Law of Mass Action to this chemical equation we should expect that if *Y* is in excess,

$$\begin{array}{l} \frac{d[P_{G}]}{dt}\;=\;KH([T_{G}])(t)[mRNA(P_{G})](t)\\ \;\;\;\;\;\;\;\;\;\;\;\;\;\;\;\;\;\;-\mu [P_{G}](t). \end{array}$$

where [*mRNA*(*P**_G_*)] is the cytoplasmic concentration of *mRNA* that codes for *P**_G_* and where we have included an additional decay term *μ*[*P**_G_*] on the right of this last equation. The Heaviside factor *H*([*T**_G_*](*t*)) reflects the fact that it is the transcription factor, *T**_G_*, that initiates the *mRNA*(*P**_G_*) production. (The factor *n* is included in the constant *K* in this last equation.)

Because the available *mRNA* for each gene is proportional to the number of cells whose genes express it, we can write, for some constants *k**_G_**, μ**_G_*

(A.2) $$\begin{array}{l} \frac{d\;[P_{G}]}{dt}=k_{G}H\;\left(\left[T_{G}\right]\right)\;(t)\;N\;(t)\\ \;\;\;\;-\mu_{G}\;[P_{G}]\;(t). \end{array}$$

Depending on other underlying processes within the cell, this rate equation may be subject to further modification. For example, one might have two proteins, one of which functions as an enzyme for the degradation of the other. For example, if protein *P**_G_1__* functions as a regulator for protein *P**_G_* with *K**_cat_*, *K**_m_* as kinetic constants, then the equation that replaces (A.2) will be

(A.3) $$\begin{array}{l} \frac{d[P_{G}]}{dt}=k_{G}H\;\left(\left[T_{G}]\;(t\right)\right)\;N\;(t)-\mu_{G}[P_{G}]\;(t)\\ \;\;\;\;-\frac{K_{cat}\;[P_{G}]\;(t)\;\left[P_{G_{1}}\right]\;(t)}{K_{m}+[P_{G}]\;(t)} \end{array}$$

**Remark 3.** In (A.2), (A.3) the assumption was that the concentration of cell nutrients [*Y*] is in excess. This need not be the case, especially in situations that lead to tumor cell necrosis. Therefore, one may need to modify (A.2), (A.3) by replacing *k**_G_* by *k**_g_* *Y/*(*K**_m_* *+ Y*), which would then reflect the fact that the rate of transcription is also proportional to the level of resources available:

(A.4) $$\begin{array}{l} \frac{d\;[P_{G}]}{dt}=\frac{k_{g}H\;([T_{G}])\;Y\;(t)}{K_{m}+Y\;(t)}N\;(t)\\ \;\;\;\;-\mu_{G}\;[p_{G}]\;(t) \end{array}$$

or

(A.5) $$\begin{array}{l} \frac{d[P_{G}]}{dt}=\frac{k_{g}H\;([T_{G}]\;Y\;(t)}{K_{m}+Y\;(t)}N\;(t)\\ \;\;\;\;-\mu_{G}[P_{G}](t)\\ \;\;\;\;-\frac{K_{cat}[P_{G}](t)[P_{G_{\;1}}](t)}{K_{m}+[P_{G}](t)}. \end{array}$$

The rate law for nutrient consumption must also be introduced. It will take the form:

(A.6) $$\begin{array}{l} \frac{d[Y]}{dt}=-\sum_{G}\left[\frac{H\left(\left[T_{G}](t\right)\right)k_{G}[Y](t)}{K_{G}+Y(t)}(t)\right]\\ \;\;N(t)+S_{r}. \end{array}$$

where the sum is taken over all genes that are expressed as proteins. The term *S**_r_* includes both tissue sources of nutrients and diffusive effects. For example, if the distribution of nutrients is not spatially homogeneous, then

(A.7) $$S_{r}=D_{Y}\text{Δ}\;[Y]+Y_{r}\;(x,y,t).$$

where *Δ* denotes the Laplace (diffusion) operator (*Δ*[*Y*] = div(grad [*Y* ]). The term *Y**_r_* (*x*, *y*, *t*) includes any tissue generated nutrients that contribute to the cellular events described above. Boundary conditions for (A.7) will include capillary supplied nutrients that move from the vasculature into the ECM.

## Appendix B. Parameter values and Normalizing Constants

The determination of appropriate biological constants from the literature is notoriously difficult. Not only are *in vivo* values often different from *in vitro* values, but very likely the former are unknown. When the constants for a given protein are known, the protein itself may function entirely differently *in vivo* than *in vitro*. That is to say, different cell types can respond to the same growth factors in different ways, and the response of a single cell type can be influenced by its environment, including the cells that surround it. Thus, the responses to a growth factor observed *in vitro* of a particular cell type grown as a mono culture are not necessarily the same as for the same cell type in tissues *in vivo* where its neighbors are not of the same cell type [40]. Thus, the task to find good values for the model is daunting, to say the least. We took a pragmatic approach, using available literature values where possible and trial values, when no values were available to us.

### Notes on Parameter Values

In the simulations, the spatial domain *D* was assumed to be a sphere of radius *R* centered at the origin, and all variables were assumed to spatially depend only on the radial variable, *r*, the distance to the origin. Table 4 contains a list of the model parameters that were used in the simulations. The basic units of measurement are millimeters (1 *mm* = 10^−3^ meters), hours (*h*), and micromoles per liter (1 *μ*M = 10^−6^ moles/L). Cell densities have the units of cells per liter. Although many of the values are based on data reported in the literature, it was not possible to find literature supporting all of the values. The values in the table with no supporting data are described as simulated values. Several of the parameter values were taken from [43], where supporting references can be found.

Unit conversions were used to determine some of the parameters from available data. To convert molecules per unit volume to micro moles per liter, we used the fact that one milliliter equals one cubic centimeter. Thus 1*L* equates to a volume of 10^15^ *μm*^3^. Hence *N* molecules per *μm*^3^ equates to *N* × 10^15^ molecules per liter. Dividing by Avogadro’s number gives (*N*/6.02) × 10^−8^ moles/L = (*N*/6.02) × 10^−2^*μM*. Similarly, to convert molecules per cell to *μM*, we estimated that the volume of a typical cell is 10^3^*μm*. This value is consistent with the available data for endothelial cells (cf. [43]). Using this value, *N* molecules per cell translates to *N* × 10^−3^ molecules per *μm*^3^, or (*N*/6.02) × 10^−5^*μM*.

The kinetic rate constants for p53, Sp1 and GF were computed from translation rates of mRNA and the number of amino acids in each protein. Assuming 10 amino acids are translated per second and there are *M* amino acids in the protein, a cell containing *N* mRNA’s would produce 10*N*/*M* protein molecules per second, or (3.6 × 10^4^)*N*/*M* molecules per cell per hour. Using one of the unit conversions described above, this results in (3.6/6.02) × *N/M* × 10^−1^ *μM/h*. Estimating that there are 750 mRNA’s per cell of p53, that contains 393 amino acids, this calculation gives a rate *k**_p_* = 0.114*h*^−1^. The amino acid counts: 696 for Sp1, 191 for GF; and the estimates: 750 mRNA’s per cell for Sp1, 1000 mRNA’s per cell for GF, yield the entries for *k**_s_*, *k**_v_* in the table.

Estimates of half-lives τ were used to determine decay rates *μ* = ln 2/*τ*. The half-life of p53 was estimated to be 30 minutes, yielding *μ**_p_* = 1.4/*h*. The half-life of GF is on the order of 20 minutes so that *μ**_v_* = 2.08/*h*. In [23], a half-life of VEGF in blood was reported as 33.3 ± 13min.

The carrying capacity for fibronectin was obtained from extrapolation and unit conversion from data taken from [5] and [67]. According to both, the maximum density of a single layer of fibronectin molecules is 3 × 10^3^*μm*^2^. Extrapolating, by using a molecular width of 35 angstroms, yields 8.57 × 10^5^ molecules per *μm*^3^, which converts to 1.4 × 10^3^*μM.* The kinetic rate constant is the same as in [43].

The carrying capacities for the cell densities obtained from extrapolation and unit conversion were based on the estimate of 10^3^ *μm*^3^ for the from data taken from [5] and [67]. According to volume of a typical cell. Since 1*L* = 10^15^ *μm*^3^, this estimate yields a carrying capacity of 10^12^ cells per liter. *D**_B_* and *D**_T_* were assumed constant, with values the same as those used in [43]. Both cell types were assumed to divide once every 48–60 hours (cell doubling time) in response to growth factor. A value of 56 hours was used as a doubling time to determine λ*_B_*, λ*_T_*. The apoptosis rates used are similar to those used in [43], and determine normalized constants that are near the carrying capacities.

### Sensitivity Functions

For the simulations, we chose to define the sensitivity functions rather than the probability transition functions. The flux terms were taken as

$$\begin{array}{l} {\vec{J}}_{B}\;=\;D_{N}\;[-\frac{\partial \eta_{B}}{\partial r}\;+(\xi_{1}(m)\frac{\partial m}{\partial r}\\ \;\;\;\;\;\;\;\;\;\;\;+\;\xi_{2}(f)\frac{\partial f}{\partial r}\;+\;\xi_{3}(Z)\frac{\partial Z}{\partial r})\eta_{B}]\\ {\vec{J}}_{T}\;=\;D_{N}[-\frac{\partial \eta_{T}}{\partial r}\;+\;(\xi_{1}(m)\frac{\partial m}{\partial r}\\ \;\;\;\;\;\;\;\;\;\;+\xi_{3}\frac{\partial Z}{\partial r}\eta_{T})] \end{array}$$

The sensitivity functions were defined by

$$\begin{array}{l} \xi_{1}(m)\;=\;-\alpha (m\;-\;m_{e}(r)),\\ \xi_{2}(f)\;=\;-\beta (f\;-\;f_{e}(r)),\\ \xi_{3}(Z)\;=\;-\gamma Z, \end{array}$$

with *α* = 1.25 × 10^+1^, *β* = 7.5 × 10^+1^, and γ = 2.5 × 10^+1^.

The sensitivity functions having the form ξ(*c*) = –*α*(*c – c*_0_) correspond to movement up the gradient when the concentration *c* is below the level *c*_0_, and down the gradient when the concentration *c* is above the level *c*_0_. Implicitly, *c*_0_ is a desirable level of the concentration. Assuming *c*_0_ is a constant, the corresponding probability transition function is τ(*c*) = exp(–*α*(*c* –*c*_0_)^2^/2), a Gaussian with mean *c*_0_ and variance *σ*^2^ = 1/*α*. In the simulations, we chose to let the desirable levels be determined by the computed equilibrium values, so that they depended on location.
